# Observations on Gun-Shot Wounds

**Published:** 1842-01-22

**Authors:** W. Poyntell Johnston


### MEDICAL EXAMINER.

NEW SERIES.

No. 4.] PHILADELPHIA, JAN. 22, 1842. [Vol. I.

### ORIGINAL COMMUNICATIONS.

*Observations on Gun-Shot Wounds.* By W. Poyntell Johnston, M. D.

In the case of gun-shot wound last presented, (see No. 1,) I wished chiefly to direct attention to the gravity of the prognosis, in a wound which could scarcely have failed to injure the brachial artery, which certainly interested the pleura and peritoneum, the lungs, liver, and diaphragm, and in which, from the symptoms, there existed a reasonable apprehension that even the stomach had not escaped. The patient promptly recovered, and the progress of cure was unaccompanied by any very severe symptoms; inflammation of the different organs interested supervened, as was anticipated, but *almost without pain*; a fistulous communication was established between the liver and lung, producing a copious expectoration of bile, which spontaneously closed, and the patient was discharged, perfectly cured, with the exception of the paralysis of the radial nerve, which was, of course, irremediable.

The remaining cases are presented in a much more condensed form, those points only being noted which are peculiar in each case, or which offer a contrast with the first.

Case II.—*Gun-shot wound of Forearm—favourable prognosis—profuse suppuration—purulent resorption—pneumonic expectoration—typhoid symptoms—sudamina—expectoration of the matter of metastatic abscesses—expectoration of gangrenous matter—death. Autopsy—metastatic abscess of lungs with circumscribed gangrene—decomposition of the blood—injection, with apparent false membrane of the cardiac extremity of the aorta—*NO INFLAMMATION *of the veins of the arm, which contain* NO PUS.

John Johnson, æt. 28, not intemperate, was gunning in a boat on the 6th September, 1835. On leaving the boat, his fowling piece, which was directed towards him, rolled to one side; in order to arrest it, he seized the barrel with his right hand in such a manner that the muzzle was in contact with the anterior part of the forearm, when it exploded, and the entire load, passing upwards and backwards, traversed the forearm, and escaped below the olecranon process; he did not fall; complained of considerable pain, and lost very little blood. He was carried three miles in the boat to his residence, where simple rags were applied to the wound, and, eighteen hours after the accident, was admitted into the Pennsylvania Hospital. For the two following days the treatment consisted in the application of poultices to the wound, which was extremely painful, and in low diet.

On the fourth day I entered as acting house surgeon, and saw him for the first time. The following was his actual state. Face flushed, (his wife attributes this to the action of the sun on a naturally florid complexion;) he slept well during the night, and complains of no uneasiness except in the wound, the pain of which has been augmenting since the accident; pulse at the wrist almost as well marked as in the opposite member, rather full, eighty; tongue natural; appetite diminished; abdomen indolent; bowels free; intellect unimpaired; thorax well developed; has never had cough, or pain in the chest; respiration healthy throughout, (carefully examined by Dr. Gerhard.) Half way between the wrist and elbow, opposite the anterior surface of the ulna, exists a wound of the size of a half dollar, with ragged and whitish edges, filled with a greenish black slough, which rises to a level with the surrounding skin; the upper edge of this wound is elevated, while that towards the wrist is depressed. On the posterior face of the forearm, just beneath the olecranon, is seen a crescent-shaped laceration, of which the concavity is turned towards the radius; it is about four inches in length, the edges are separated, are very ragged, and the whole of the interior is covered by a greenish black slough, similar to that already mentioned; at the bottom and to the inside of this wound the ulna is exposed for about an inch; the surface is smooth, and appears to have served as a director for the escape of the load; the anterior part of the forearm is swollen, but the skin is not tender; the arm above the elbow is reddish posteriorly, and anteriorly presents various shades of green and yellow.

The prognosis, in regard to *the life* of the patient, was decidedly favourable: a young, temperate, healthy, and robust man, had received a gun-shot wound of the forearm, involving no important parts; the articulation was apparently uninjured; the bones were not fractured; the arteries had escaped; and, on the fourth day after the reception of the injury, the general health was unimpaired, the arm but moderately swollen, the pulse on the injured side distinct, and only at eighty; in a word, the only uneasiness experienced was the pain caused by a lesion of some of the filaments of the brachial plexus.

Had the olecranon process been injured, and the cavity of the elbow-joint exposed, it is a question whether a prognosis founded upon probabilities, would not still have been in the patient's favour. The following is a case in point:

—— Bertier, æt. 45, formerly a soldier in the army of Napoleon, received, in 1813, a gun-shot wound, similar to the above, involving, however, the olecranon; from that period a fistula remained at the posterior part of the elbow joint, which at intervals became swelled and painful; he nevertheless continued his work, as ditcher, up to the commencement of 1833. At this period, twenty years after the injury, the swelling increased, and the severe pain prevented the motions of the joint. He entered the hospital, St. Louis, April 12, 1833, and came under my charge as *Externe*. The elbow was slightly swelled, the motions painful; the disease was regarded as a white swelling, and amputation was performed the 16th. Death took place on the 29th, from purulent resorption with metastatic abscesses of the liver.

This short abstract is here introduced with a view of exhibiting upon what reasonable grounds a favourable prognosis was formed in the case under consideration. After twenty years of hard labour, Bertier died of the amputation, not of the gun-shot wound.

This case will again be referred to in elucidation of another point.

But to return to the case of Johnson. On the 6th day the arm became more swelled, and at half-past nine he was seized with a *very severe chill*, which lasted half an hour, and was immediately succeeded by profuse perspiration. (Eighty leeches to arm; cold poultices.)

On the 7th day the arm, which was twice the size of the opposite member, offered an erysipelatous tint, which gradually disappeared under cold applications; the strength of the patient decreased.

On the 10th day there had been no return of the chill, but the patient suffered from continued and profuse perspiration; the whole of the right arm, the chest, and the abdomen were covered with sudamina; the countenance anxious; the wounds granulating; the prostration marked. (Poultices to wounds. Porter, eggs, and milk for diet.)

The sudamina and sweats continued till the 16th day, with increasing feebleness, when they were replaced by a diarrhœa, which was checked by opium injections. The strength and appetite improved, but the suppuration became very profuse.

On the 21st day, on removing the dressings, the arm was found floating in the profuse discharge from the wound in the elbow. On pressing the forearm along the track made by the load, a large quantity of thick pus was evacuated, and, on pressing the inside of the arm, about half a pint more of fluid pus escaped from an abscess which was situated beneath the brachial aponeurosis; finally, on making compression over the external condyle, a small quantity of serous fluid oozed from the wound near the elbow. General health much improved; no sudamina; little pain. Pulsations of radial artery distinct, although the hand was very œdematous. The patient was not emaciated; the countenance was coloured, and free from anxiety; no return of chill; respiration easy; no no cough, or pain in the chest; the patient felt stronger than since his entrance.

(Generous diet, port wine, bandage of Scultetus.)

He continued improving in strength and general health until the 23d day, when, at 10 A. M., he was again seized with a *violent chill*, which lasted a quarter of an hour, and was followed instantaneously by a profuse perspiration, which lasted till night.

On the following day (the 24th since the accident) the countenance was anxious; the speech hesitating, as he thought from weakness; the wounds more animated; *the discharge not diminished*, free from odour. General health as before, with the exception of feebleness. (The whole limb was enveloped in a bandage drawn tolerably tight, leaving the wounds exposed, to which poultices were continued.) He sweated profusedly during the whole night, and on the morning of the 25th day. The mattrass, pillows, blankets, &c., were completely soaked. A vapour arose from his body, when uncovered, which was very perceptible at some distance. The pulse was full, not accelerated. The countenance red, not emaciated; the discharge undiminished in amount and unaltered in quality. No diarrhœa. Respiration easy. Auscultation over the anterior portion of the chest revealed no alteration. (Wine; bitter tea.) The suppuration continued profuse and free from odour on the following day, and the wounds were filled with bright red granulation. On the reapplication of the bandage there was a gush of about six ounces of arterial blood from the wound near the elbow; the bandage was immediately removed, but the hæmorrhage had ceased; the pulsations of the radial artery were as distinct as before. A tourniquet was loosely applied; poultices to the wounds; and the bandage was omitted. A *sense of chilliness* was experienced at 10½ A.M., which lasted a quarter of an hour, and was immediately followed by profuse sweating. Up to the 28th day the perspiration was continual, and the discharge from the wounds undiminished; the countenance became pale and anxious; the granulations lost their florid colour, and were covered by a whitish slough; the exposed portion of the ulna assumed a dark-brown colour, and the hæmorrhage did not recur.

On the 29th day, after a good night, he expressed himself as feeling better than he had done since his admission. The face was again coloured; pulse full, about 100; the discharge continued profuse, more fœtid, and was mixed with *numerous bubbles of air.* No return of chill or hæmorrhage. No oppression of chest or cough. Bowels free. (Wine; soups.) His general health and strength for the two following days improved rapidly. On the 31st day he complained only of slight pain in the epigastrium, and a *great desire to cough,* to which he was afraid to yield. At half past one *he was seized with a severe pain in the left side,* and at 4, A.M., yielding to the desire to cough, he expectorated a single *well marked pneumonic sputa.* Examined by Dr. Gerhard, he presented obscurity on percussion on the left side of the back as high up as the scapula. Egophony over the same space, and a subcrepitant rhoncus at the base of both lungs posteriorly.

From this period the pain in the side diminished, and finally disappeared. The pain in the epigastrium continued, but no longer prevented the cough, which was accompanied always by the pathognomonic sputa of pneumonia. The pulse became more feeble, ranging from 115 to 120; the strength declined. On the 33d day he expectorated a moderate quantity of viscid, rust-coloured matter, in the midst of which was found a single, tolerably large mass of a greenish yellow colour, similar to partially concrete pus, *and perfectly resembling the matter found in metastatic abscesses of the lungs when they begin to soften.* The discharge from the wounds continued unabated. (Elixir vitriol; bitter tea; port wine; emolient enemeta.)

The general health improved, although the cough and expectoration of pneumonic sputa, mixed with concrete pus, continued up to the 36th day, when he was seized at noon with a *sudden and very*

*violent chill,* with chattering of the teeth, which lasted twenty minutes, and was followed immediately by profuse sweat, which continued almost without intermission for some days; the expectoration gradually lost its pneumonic character, and *resembled more and more the matter found in metastatic abscesses when perfectly softened.* The prostration increased. Port wine, beef tea, and eggs were taken without relish, but without disgust; the suppuration continued undiminished. Milk punch and elixir of vitriol were administered, with a slight increase of strength. On the 42d day the expectoration *became slightly fœtid;* and on the 43d day, in the evening, there was a sudden aggravation of all the symptoms; great dyspnœa; cough in paroxysms; the expectoration became very difficult, was still purulent, but of a *darker colour,* and *exhaled a well marked gangrenous odour.* This was accompanied by a full and quick pulse; a hot and moist skin; delirium and subsultus tendinum. On the following day the prostration increased; *patches of gangrenous matter* were found in the sputa. Musk, and milk punch were freely administered, and towards evening he became calm.

On the 45th day the countenance was improved, but the intellect was confused. A low muttering delirium supervened. The cough was rather harassing; expectoration moderate, fluid, purulent, having the odour of musk; suppuration very profuse.

On the 46th day the countenance became altered; the eyes dim; skin warm and moist; pulse fluttering, about 140; dyspnœa; cough frequent and harassing; expectoration difficult, small in quantity, purulent, exhaling only the odour of musk. During the day the prostration increased, and death occurred at 1 P. M.

### *Autopsy, twenty hours after death.*

*External Appearances.*—Emaciation; no rigidity of limbs; cranium not opened.

*Thorax.*—Old strong pleuritic adhesions on both sides; recent adhesions, with purulent false membrane between the pleura and diaphragm on left side; effusion of a tolerable quantity of serum into the cavities.

*Right Lung.*—Slight interlobular emphysema; tissue generally pale and crepitant. At the base of the lung, immediately above the pleura, exists an abscess of the size of a hen's egg, or rather larger, very infractuous, surrounded completely by false membrane, and enclosing a moderately sized dark brown slough, and filled with the same purulent matter which had been expectorated during life. Several of the larger bronchial tubes opened directly into this abscess, one of which was sensibly dilated. The bronchial mucous membrane was cut off abruptly at the opening, and throughout the rest of the tube was red and slightly thickened. The tissue surrounding this abscess was, for about a line, red and indurated, but still contained air; and immediately around this induration the tissue was slightly engorged; elsewhere it was perfectly crepitant, and in no part completely hepatised.

The left lung presented a perfectly similar alteration, both in size and situation; the slough contained was, however, perfectly black, and emitted a gangrenous odour, notwithstanding the strong smell of musk exhaled by the whole body. The slough adhered in some parts to the false membrane bounding the abscess, and in these points presented a brownish red colour. The pus contained was of the same nature as in the opposite side; but the bronchial tubes opening into it more obliquely were not sensibly dilated, but were thickened and reddened throughout the lung. The parenchyma surrounding this abscess presented the same characters as in the opposite lung, and in the indurated tissue immediately above the abscess was found another smaller cavity, about the size of a bean, perfectly isolated, surrounded by a thin false membrane, and containing a blackish fluid. The rest of the lung was healthy, with the exception of a slight interlobular emphysema.

*The Pericardium* contained about a pint of serum.

*The Heart* large, pale, flabby; tissue softened, permitting the fingers to penetrate it with tolerable facility. No coagula in any of the cavities, but a small quantity of red and very watery blood. The semilunar valves, and the adjoining portions of the aorta and of the lining membrane of the heart, were covered by a thin transparent membrane, not thicker than gold beater's leaf, which could be detached with moderate ease by scraping.

The lining membrane beneath this was moderately injected, except over one of the semilunar valves, where it was of a vivid red, and in this spot the apparently false membrane covering it was thick, opaque, and adhered so strongly that it could with difficulty be torn off.

*Stomach* large; mucous membrane pale and consistent.

*Liver* pale and firm; examined by fine slices.

*Spleen* of natural size and firmness, of a reddish gray colour generally; but presenting at its point externally a patch about the size of half a dollar, of a bluish tint, which does not extend into the parenchyma.

*Kidneys* perfectly healthy.

Veins of the right arm perfectly sound, lining membrane pale and firm; large vessels of thorax healthy, except aorta near the heart.

